# HCV seroconversion in a cohort of people who use drugs followed in a mobile harm reduction unit in Madrid: Breaking barriers for HCV elimination

**DOI:** 10.1371/journal.pone.0204795

**Published:** 2018-10-03

**Authors:** Jorge Valencia La Rosa, Pablo Ryan, Alejandro Alvaro-Meca, Jesús Troya, Guillermo Cuevas, Jorge Gutiérrez, Santiago Moreno

**Affiliations:** 1 Unidad Móvil de Reducción del Daño, Subdirección General de Asistencia en Adicciones, Madrid, España; 2 Medicina Interna, Hospital Universitario Infanta Leonor, Madrid, España; 3 Departamento de Bioestadística, Universidad Rey Juan Carlos, Madrid, España; 4 Organización no gubernamental “Madrid Positivo”, Madrid, España; 5 Servicio de Enfermedades Infecciosas, Hospital Universitario Ramón y Cajal, Universidad de Alcalá, IRYCIS, Madrid, España; University of Cincinnati College of Medicine, UNITED STATES

## Abstract

**Background and aims:**

Harm reduction strategies have been shown to decrease the incidence of human immunodeficiency virus (HIV) infection in people who inject drugs (PWID), but the results have been inconsistent when it comes to prevention of hepatitis C virus (HCV) infection. We aimed to examine the rate of HCV seroconversion among people who use drugs (PWUD) followed at a mobile harm reduction unit (MHRU) to evaluate if a low-threshold methadone substitution program (LTMSP) is associated with a low HCV seroconversion rate and subsequently identify barriers for elimination.

**Materials and methods:**

A cohort of PWUD have been followed at a MRHU in Madrid between 2013 and 2016. Individuals who were negative for HCV antibodies at baseline and who had at least one retest for HCV antibodies were eligible. Kaplan-Meier methods were employed to estimate the global incidence density.

**Results:**

During the study period, 946 PWUD were screened for HCV at least once. At baseline 127 PWUD were negative for HCV antibodies and had at least one follow-up HCV antibodies test. The baseline HCV prevalence was 33%. After a median 0.89 (IQR 0.3–1.5) years of follow-up and 135 person-years of risk for HCV infection, 28 subjects seroconverted. The incidence density for HCV seroconversion for this sample was 20.7 cases (95% CI: 14.3–29.7) per 100 person-years. Injecting drugs in the last year was strongly associated to HCV seroconversion (AHR 15.5, 95%CI 4.3–55.8, p < 0.001). Methadone status was not associated to HCV seroconversion.

**Conclusions:**

A high incidence of HCV infection was found among PWUD at a MHRU in Madrid. In this setting opiate substitutive treatment (OST) as a LTMSP does not appear to protect against HCV seroconversion.

## Introduction

According to the World Health Organization (WHO), viral hepatitis was the seventh highest cause of mortality in 2015, being responsible for an estimated 1.3 million deaths per year from acute infection and hepatitis-related liver cancer and cirrhosis[1]). Of those deaths, approximately 30% are attributable tohepatitis C virus(HCV). World Health Organization estimates that worldwide, there were about 1.75 million new HCV infections in 2015 [2]).

It is estimated that there are 15.6 million people who inject drugs (PWID) between the ages of 15 and 64 years[3]). Globally 52.3% of PWID are HCV-antibody positive, although with substantial geographic variation. China, USA and Russia had the largest such populations[4]).New HCV cases continue to occur among PWID despite the implementation of harm reduction strategies, with an HCV incidence in this group ranging from 10 to 50 cases per 100 person-years [5–8]).There are several known factors associated with the acquisition of HCV infection, including older age, recent onset of injection drug use, sharing of syringes, engaging in risky sexual behaviors, commercial sex work, frequent injection of cocaine [9]), homelessness [10])and reporting front- and back-loading [11]).

Harm reduction programs are designed to prevent blood-borne infections transmission in PWID and include needle and syringe programs (NSP) and opiate substitutive treatment (OST) as core interventions [1]). Although they have been shown to be effective at reducing HCV seroconversion [12]), a recent systematic review found that NSP were effective in reducing HIV transmission[13]), while there were mixed results regarding a reduction of HCV infection [14]). Similarly, OST has been associated with lower relative hazards for becoming infected with HCV over timecompared to those not on OST [15]).

There are several reasons that could help explain why these interventions have not been consistently successful in decreasing the HCV incidence in PWID [14, 16]. Among others, an important reason is the ineligibility for HCV treatment of drug users in active consumption [17]) and certain populations at risk for HCV infection (e.g., incarcerated, homeless, and uninsured persons) with limited or no access to care [18]), which could likely be the focus of new HCV infections. The low-threshold methadone substitution programs (LTMSP) have been designed for these specific populations. They are flexible intervention programs that give OST and treat a marginalized population of people addicted to heroin with unstable lifestyle, who would not have access to regular programs[19]).

The specific objective of this study was to measure the HCV incidence and to examine the factors associated with HCV seroconversion among PWUD activelydrugs users enrolled in a MHRU in Madrid, with the goal of assessing if a LTMSP is a protective factor in HCV seroconversion rate in the times of the DAAs.

## Materials and methods

For the present observational study, we pooled data from a cohort of PWUD who actively consumed heroin and/or cocaine, either smoked or injected, and were being followed at a MHRU located in the outskirts of Madrid, Spain.Data are available from the Subdirección General de Adicciones (Madrid, Spain) Institutional Data Access for researchers who meet the criteria for confidential data.When entering the MHRU, clients sign different documents, which include informed consents for blood tests, standard follow-up at the Unit, and inclusion of information in a database for purposes of analysis. The database is anonymized with an alphanumeric code unique for each client, so that no person can be identified and linked to the registered information. In these circumstances, no additional approval from an Ethics Committee was required.

The MHRU attends PWUD actively street outreach who have limited access to standard healthcare. This population consumes heroin and/or cocaine usually mixed in different proportions with one another. Both drugs can be used via smoked, either through pipes or aluminum foil, or injected. This information was collected through direct report to study personnel when they request the injection equipment (needles and syringes) at the MHRU.Participants report comorbidities, such as blood borne virus infections, skin and soft tissue infections, overdoses, emergency derived aggressions, impaired physical conditions, and poor access to standard medical care. Also, they maybe psychologically challenged due to mental illness associated with drug use, socially excluded, and may have multiple criminal records and behaviors related to thefts.

The study was designed to estimate the time to HCV seroconversion of PWUD actively followed in our MHRU in the period from January 2013 to December 2016. This MHRU offers addiction treatment, directly observed treatment (DOT) of chronic diseases, sex and blood borne infections counselling and testing, risk-reduction counselling, social services, primary medical care, condoms/lubricants, clean injection equipment, sterile needle and syringes, all free of charge. Also, OST is prescribed as an LTMSP. All individuals recruited during the study period were eligible for the analysis of baselineprevalence of HCV antibodies, whereas only individuals who were HCV-negative at baseline, and who had at least one follow-up visit (to re-test for HCV infection) were eligible for the analysis of HCV incidence density.

An HCV enzyme-immunoassay [EIA] and rapid tests were used as screening. Individuals with an initial positive HCVantibodies test were considered for the prevalence calculation and excluded from the incidence density analysis. Seroconversion was considered if HCV antibody changed from negative to positive during the study period. Baseline characteristics were collected for analysis of HCV seroconversion predictors in seroconverters and non-seroconverters.

### Statistical analysis

Data for the analysis was collected from the MHRU database that registered the unit's activity between 2013 and 2016. As an initial step, Kaplan-Meier methods were employed to estimate the overall incidence density and incidence density according to methadone status; 95% confidence intervals were calculated with normal approximation given the frequent events. The date of HCV seroconversion was estimated as the midpoint between an individual’s last negative and first positive HCV antibodiestest. Participants remaining persistently HCV negative were censored at the time of their most recent available HCV antibodies test result prior to December 2016.

We also calculated the unadjusted relative hazard of HCV seroconversion using Cox proportional hazard regression, and stratified for methadone status, to assess the independent effect of an LTMSP on time to HCV seroconversion. Methadone status was determined as positive or negative according to the administration of methadone in the period between the first and last HCV serology. We also considered secondary variables that might potentially confound the relationship between the methadone status variable and the outcome. The following variables were included in the adjusted analysis: gender (male vs. female), age (per 10 years older), nationality (Spanish vs. non-Spanish) and the use of injected drugs during the last year (yes or no).

Analysis were conducted using R Software, the threshold for statistical significance was set at p< 0.05. All p-values were two sided.

## Results

During the study period, 946 PWUD were seen at the MHRU and had performed at least an HCV antibodies test as part of the individual, initial intervention in our MHRU. HCV and HIV antibodies prevalence of the initial sample were 33.3% and 4.8%, respectively.

**Fig 1:**
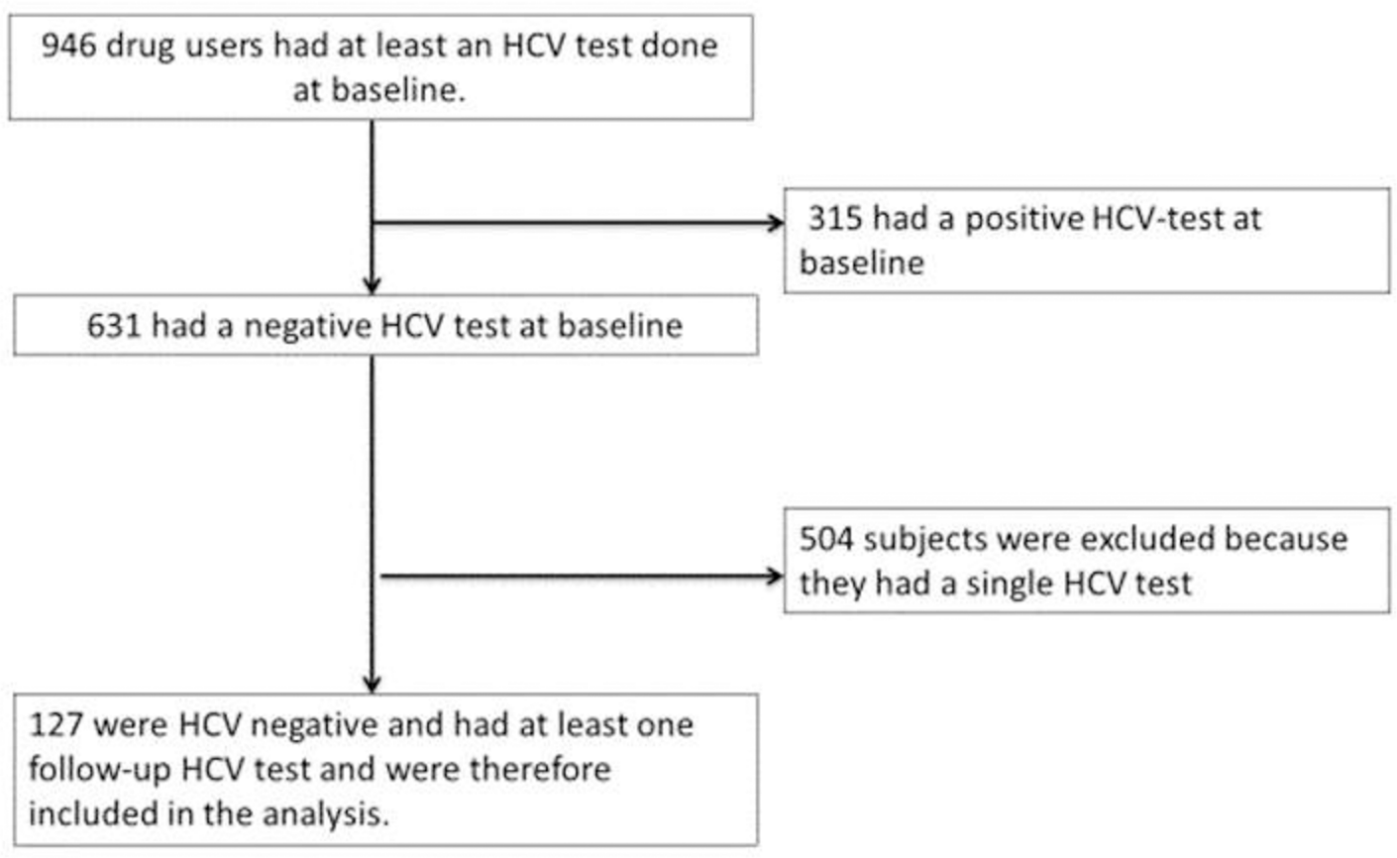
Flowchart of the study population.

504 subjects were excluded because they had a single HCV antibodies test, and 315 due to being HCV-infected at the first visit Fig 1. At baseline, 127 PWUD were HCV negative and had at least one follow-up HCV antibodies test and were therefore included in the analysis of HCV incidence density. HIV positive individuals were not excluded from the study; however, all 127 PWUD were HIV-seronegative at baseline.

Among the 127-baselinenegative HCV PWUD, the mean age was 41.3 (SD ±8.5), 72 (59%) were male, 99 (81.1%) were Spaniards and 37 (38.5%) used injected drugs during the last year. Overall, 53 (41.7%) subjects were receiving methadone. After a median 0.89 (IQR 0.3–1.5) years of follow-up and a total of 135.1 person-years of risk for HCV infection, 28 subjects seroconverted for HCV. Also, one HIV seroconversion was found. Although the information is not routinely collected in all PWUD, all HCV seroconverters drug users reported to be heterosexual and denied the use of chemsex. Baseline characteristics of the PWUD are shown in Table 1.

**Table 1:** Baseline characteristics of the population of study and HCV incidence.

		HCV incidence density
	N (%)	Rate per 100 PY; 95%CI
**Characteristics**		
Age*	** **	** **
Mean (SD)	41.3 (±8.5)	unreported
Sex*	** **	** **
Male	72 (59%)	18.5 (9.2–27.9)
Female		28.1 (12.8–43.4)
Spanishnationality*	** **	** **
Yes	99 (81.1%)	25.6 (15.6–35.6)
Not		10.2 (0.1–21.8)
**Methadone treatment**	** **	** **
Yes	53 (41.7%)	18.8 (9.3–28.4)
Not		24.1 (11.0–37.2)
Injecting drug users**	** **	** **
Yes	37 (38.5%)	72.5 (43.5–100.6)
Not		4 (0.1–8.6)

Note: HCV, Hepatitis C virus; SD, Standard deviation; PY, person-year

*Age, sex and nationality are missing for 5 participants

** last 12 months; date is missing for the 31 participants

**Fig 2:**
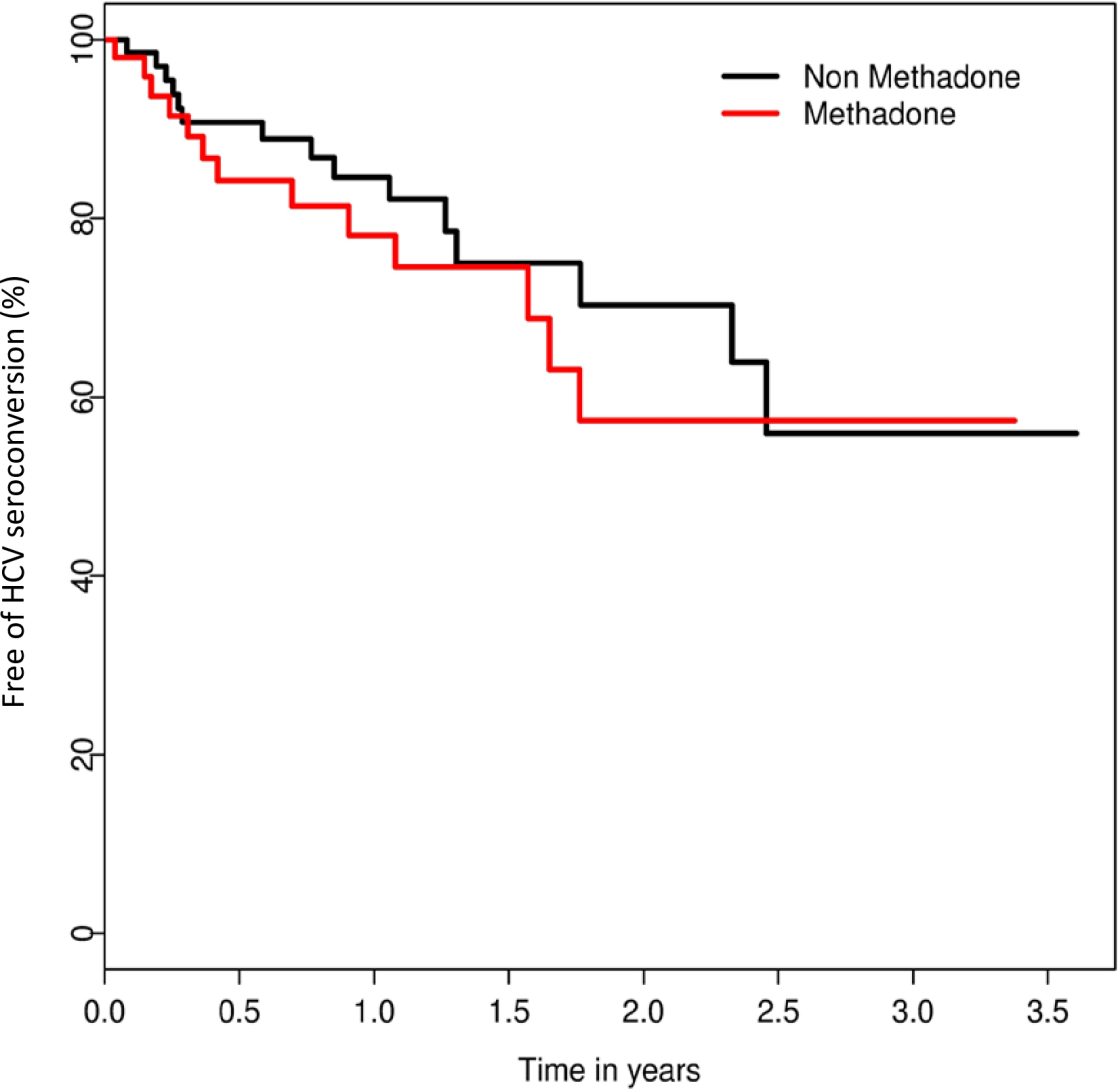
Kaplan- Meier graphs of time to HCV seroconversion stratified by methadone status at baseline, among drug users followed at a mobile harm reduction unit, Madrid, Spain. 2013–2016.

The incidence density of HCV seroconversion for the entire sample was 20.7 (95% CI; 14.3–29.7) cases per 100 person-years. Stratified by methadone status, the incidence density rates of HCV infection were as follows: 24.1 (95% CI; 11.0–37.2) cases per 100 person-years among participants with a negative methadone status, compared to 18.8 (95% CI; 9.3–28.4) cases per 100 person-years among those with positive methadone status (p = 0.26) Fig 2. Table 2 shows the results of the unadjusted and adjusted Cox proportional hazard regression analyses of the time to HCV infection for baseline characteristics. In unadjusted analysis, the age (HR = 0.91 [95% CI: 0.86–0.96], p = 0.001) and the use of injected drugs (HR = 18.94 [95% CI: 5.59–64.2], p < 0.001) were positively associated with time to HCV seroconversion. In the adjusted analysis, after adjusting for sex, age, nationality and methadone status, the use of injected drugs in the last year was positively associated with time to HCV seroconversion (adjusted hazard ratio [AHR] = 15.53 (95% CI: 4.3–55.9) p < 0.001). The methadone status was not associated with time to HCV seroconversion in the bivariable or multivariable analysis.

**Table 2:** Cox proportional hazards analyses of factors associated with time to HCV seroconversion of our study sample.

Variables	Unadjusted HR (95%CI)	p value	Adjusted HR (95%CI)	p value
**Age (per 10- year older)**	0.91 (0.86–0.96)	0.001	0.96 (0.90–1.03)	0.26 (NS)
**Sex**				
Male	0.67 (0.31–1.41)	0.29	0.74 (0.32–1.70)	0.48 (NS)
Female	1			
**Nationality**				
Spanish	2.51 (0.75–8.32)	0.13	1.76 (0.47–6.5)	0.39 (NS)
Non- Spanish	1			
**Methadone**				
Yes	1.26 (0.60–2.66)	0.53	1.37 (0.58–3.22) p 0.47	0.47 (NS)
Not	1			
Injecting drug users*				
Yes	18.94 (5.59–64.2)	< 0.001	15.53 (4.31–55.89)	< 0.001
Not	1			

Adjusted covariates include age, nationality, sex, methadone and injecting drugs use

Note: HR, hazard ratio; CI, confidence interval; HCV, hepatitis C virus; NS, non- significative.

* in the last year

## Discussion

In this study, we found that the HCV incidence density forPWUD actively followed in a MHRU of Madrid (Spain) between January 2013 and December 2016 remains unacceptably high despite the availability of newer antivirals and the universal treatment for HCV infection in our country. Furthermore, the use of injected drugs in the last year is an independent robust predictor of HCV seroconversion, and the OST as a LTMSP was not associated with lower HCV seroconversion rates.

The finding of a high incidence in our population is consistent with other studies conducted in Spain [6]) and in different countries in Europe [20, 21]) and North America [9, 22]), showing that HCV incidence density in recent years remains high and in all cases above 20 cases per 100 PY. Unsafe injection practices remains the main route of HCV transmission[23, 24]), although sharing injection equipment other than syringes may be an important cause of HCV transmission between PWID[7, 11, 23, 25, 26]).

Recent studies have reported that local injecting networks are the main viral reservoirs for HCV incident infections in developed countries [27]). The disorganized lifestyle associated with active consumption, the high prevalence of psychiatric illness and unstable housing may act, as well, as a barrier for this population in accessing HCV treatment and care [10]). In our opinion this injecting network might powerfully influence HCV transmission to new and younger injectors, people who recently change the route of drug administration, and previously treated patients who are currently in drug relapse (reinfections).

Of note, in the present study, OST as a LTMSP did not have an impact on HCV seroconversions. These results are contradictory with another previously published study which included only participants under age 30 [15]), and with a recent meta-analysis which reported reductions in HCV incidence in association with OST ranging from 40% to 60% [12]). However, the characteristics of the observational studies included in the meta-analysis could limit the conclusions since HCV incidence rates were rather low and inclusion criteria were heterogenous, such as participants in prison or variable degree of OST exposure and consumption of amphetamines jointly. Current studies show that cocaine and heroin have been the preferred injecting drugs in the last decade and highly predictive of HCV infection [28, 29]). Our population represents a precarious one with high consumption of injected or smoked heroin and cocaine and with shortorabsent periods of abstinence.

WHO would expect to reach a 90% reduction in new cases of hepatitis in 2030 [1]). However, the new HCV infections have not decreased in the last years and even after the introduction of DAA among PWUD, as shown in this report. Efforts should be directed to break barriers in the cascade of HCV treatment in the next years [22]) and forward individualized therapy for all people who actively inject drugs within an addiction care setting. Recently, EASL recommendations confirm that HCV treatment for PWID should be considered on an individualized basis and delivered within a multidisciplinary team setting, regardless a history of intravenous drug use and recent drug use [30]). Indeed, we think that new models of care for this population, such as the MHRUs, are the places suitable for HCV treatment and follow-up, that include linkage to specialized consultation, surveillance of adherence to HCV treatment, adverse effects monitoring and prevention of reinfection. We agree with P. Bruggmann and A. Litwin in that, a high level of acceptance of the individual life circumstances of PWID rather than rigid exclusion criteria will determine the level of success of any model of HCV management [27]).

Similar to HIV infection, the use of antiviral therapy as a method to prevent HCV transmission (Treatment as Prevention, TasP) among PWID has recently been described [31, 32]). Knowing that the efficacy of HCV therapy is similar among PWID and non-PWID [33, 34]) and that HCV reinfection rates are lower than expected [35]), TasP should be implemented without any restrictions in this population [17]). Also, some simulated HCV transmission models reveal that treating PWID and all or at least most of their contacts are an effective treatment strategy reducing the reinfection and incidence rates and combined infection[31]).

This study has several limitations. First, the present study does not include a random sample of PWID in our area. A significant proportion of PWUD attended at our MHRU never came back after their first visit, so the evaluated sample may not be representative of the population. It must be noted, however, that PWUD who return for HCV screening could be at a lower risk of HCV infection than those who are not engaged. Thus, we might have underestimated the real incidence, which would reinforce the conclusions of the study. Second, the method usedto calculate the date of HCV seroconversion may also limit the strength of the results. Although this is a frequently used method, it can be a limitation as presumably recent risk-taking behavior may prompt the service user to be retested. Third, the sample size is small, but it is still a representative sample of PWUD considering the high HCV prevalence in PWID in Spain. Fourth, the information collected on the characteristics of the population studied, including the drug injection practices and sexual behavior, were not uniformly collected at inclusion. We estimate that the impact on sexual transmission may be minimal given that the risk of acquiring HCV through sexual route in a heterosexual population is extremely low [36]). For the correct interpretation of the results it should be considered that no missing data imputation was conducted, thus those with missing data (primarily injecting drug use and sexual behavior) were omitted from the regression analysis. Fifth, the HCV RNA amplification was not performed routinely and therefore, the HCV reinfections were not reported during the follow-up. Given that up to 25% of participants may spontaneously clear the virus, a high reinfection rate could occur. Further studies could evaluate the impact of reinfections in the HCV incidence. Sixth, the methadone status was considered qualitative during the period of study; moreover, the duration, withdrawal and the adherence to methadone administration were not collected, which could lead to information bias. Also, these findings may not necessarily be generalizable to others OST, such as buprenorphine/naloxone or buprenorphine-based OST programs.

## Conclusions

The incidence of HCV remains high in PWUD actively followed at a MHRU in Madrid, despite a LTMSP and other current harm reduction strategies. In particular, OST as a LTMSP was not a protective factor to HCV seroconversion. The findings of this investigation may not necessarily be generalizable to all PWUD or who live in other communities or countries with different characteristics but it points to the need of different measures to limit the continuous spread of HCV among PWUD.
